# Structure cristalline, caractérisation spectroscopique, calcul DFT et analyse de surface Hirshfeld du perchlorate de *p*-toluidinium

**DOI:** 10.1107/S2056989017018096

**Published:** 2018-01-09

**Authors:** Meriam Ben Jomaa, Hammouda Chebbi, Noura Fakhar Bourguiba, Mohamed Faouzi Zid

**Affiliations:** a Université de Tunis El Manar, Faculté des Sciences de Tunis, Laboratoire de Matériaux, Cristallochimie et Thermodynamique Appliquée, 2092 El Manar II, Tunis, Tunisia; b Institut Préparatoire aux Etudes d’Ingénieurs de Tunis, Rue Jawaher Lel Nehru, 1089 Montfleury, Tunis, Tunisia

**Keywords:** crystal structure, organic perchlorate, DFT calculations, Hirshfeld surface, fingerprint plots

## Abstract

The synthesis of *p*-toluidinium perchlorate (systematic name: 4-methyl­anilinium perchlorate), C_7_H_10_N^+^·ClO_4_^−^, was carried out from an aqueous reaction of perchloric acid with *p*-toluidine. This compound was characterized by powder XRD, IR and UV–Vis spectroscopy. The structure was further confirmed by a single-crystal X-ray diffraction study. The crystal structure is formed by a succession of two-dimensional mol­ecular layers consisting of perchlorate anions and organic cations parallel to the (100) plane and located at *x* = 2*n* + ½ (*n* ∈ *Z*). Each mixed layer is formed by infinite chains {C_7_H_10_N^+^·ClO_4_^−^}_*n*_ parallel to the [010] direction and developing along the *c* axis, generating *R*_2_^4^(8), *R*_2_^2^(4) and *R*_4_^4^(12) graph-set motifs. The results of a theoretical study using the DFT method at the B3LYP/6–311++G(d,p) level are in good agreement with the experimental data. Hirshfeld surface and fingerprint plots reveal that the structure is dominated by O⋯H/H⋯O (54.2%), H⋯H (26.9%) and C—H ⋯π (14.3%) contacts. The studied crystal was refined as a two-component twin

## Abstract 2

A new organic perchlorate, C_7_H_10_N^+^·ClO_4_^−^, was synthesized by slow evaporation at room temperature and its crystal structure was determined. This compound was characterized by powder XRD, IR, and UV–Vis spectroscopy. The DFT optimized structure at the B3LYP/6–311++G (d,p) level was compared with the experimentally determined mol­ecular structure in the solid state. Hirshfeld surface and fingerprint plots are presented and discussed.

## Contexte chimique   

Les matériaux hybrides ont fait l’objet de nombreux travaux de recherche du fait qu’ils rassemblent les propriétés des molécules organiques et des composés inorganiques. Cette symbiose entre deux types de chimie trop longtemps considérés comme antagonistes s’hybrident à merveille et libèrent des propriétés complètement nouvelles, et ouvre un vaste champ d’investigations pour le chimiste.

Selon cette approche, les composés hybrides à base de perchlorates ont été particulièrement étudiés du fait des propriétés physiques intéressantes qu’ils présentent comme la ferroélectricité [perchlorate de pyridinium (Czarnecki *et al.*, 1994 ▸), perchlorate de pyridin-4-ylméthanaminium (Cui *et al.*, 2016 ▸), perchlorate de guanidinium (Drozd & Dudzic, 2013 ▸)] et l’optique non linéaire [perchlorate d’anilinium (Vivek *et al.*, 2015 ▸), perchlorate de *p*-nitro­anilinium (Bouchouit *et al.*, 2008 ▸), perchlorate de l-leucinium (Marchewka & Drozd, 2013 ▸)].

Dans ce contexte, nous avons tenté d’explorer les systèmes *A*[ClO_4_] (*A*: cation organique dérivant de l’anilinium), tel que de s’initier aux différentes techniques de synthèse, de caractérisation et d’analyse structurale ainsi que du calcul théorique DFT. Dans ce travail nous presontons la synthèse, les caractérisations spectroscopiques IR et UV–visible ainsi que la DRX sur poudre, la structure cristalline, le calcul DFT et l’analyse de surface Hirshfeld d’un nouveau matériau hybride à base de perchlorate et *p*-toluidinium de formulation (C_7_H_10_N)[ClO_4_] (I).
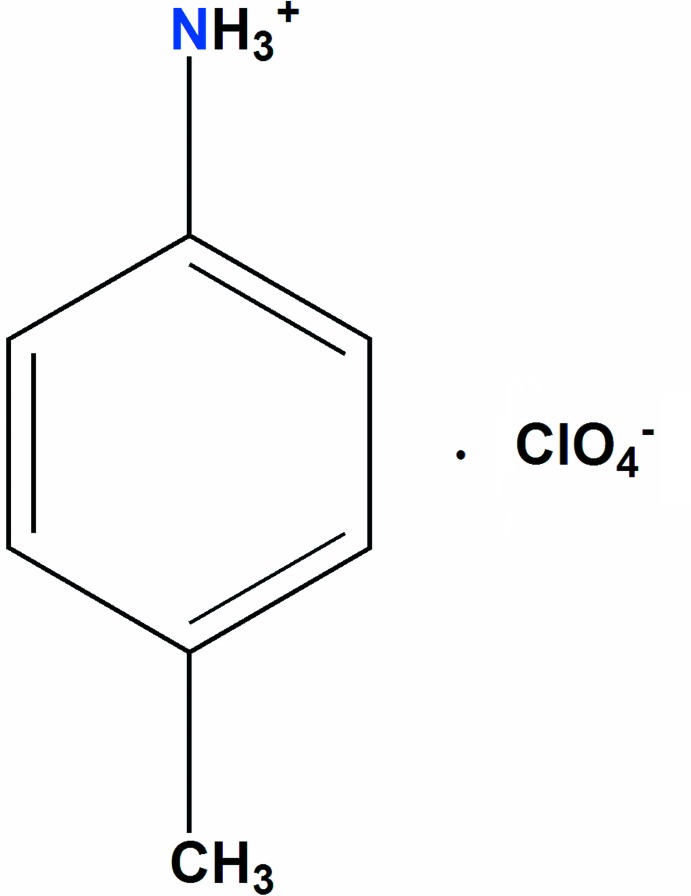


## Analyse structurale   

**Figure 1:**
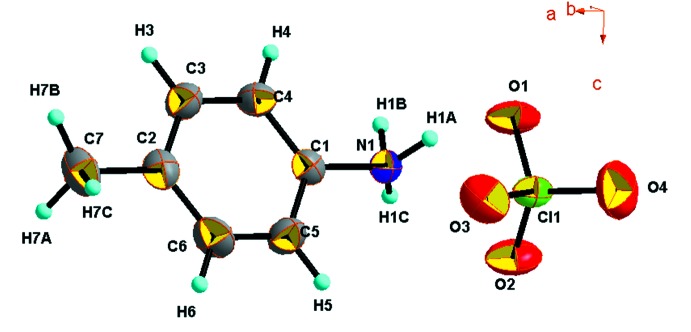
L’unité asymétrique de (I). Les ellipsoïdes d’agitation thermique ont 30% de probabilité d’existence.

L’unité asymétrique du composé (C_7_H_10_N)[ClO_4_] est formée d’un anion perchlorate [ClO_4_]^−^ et d’un cation organique (C_7_H_10_N*)^+^* (Fig. 1 ▸)*.* Tous les atomes occupent des positions générales (4*e*) et possèdent des faibles facteurs d’agitation thermique comparables à l’exception de celles des atomes d’oxygène (O1, O2, O3 et O4) du groupement perchlorate qui sont relativement élevés (*U*_eq_ > 0,1Å^2^).

L’environnement de l’atome de chlore Cl1 est tétraédrique avec une déformation considérable. En effet, les longueurs des liaisons Cl—O varient de 1,394 (5) à 1,421 (4) Å. Quant aux angles ils présentent une nette dispersion par rapport à l’angle d’un tétraèdre régulier (109,47°), puisque l’inter­valle est compris entre 105,3 (2)° et 111,7 (4)°*.* Ces valeurs sont comparables à celles du même anion associé avec d’autres types de cations (Chebbi *et al.*, 2017 ▸; Zhou & Cai, 2012 ▸; Dai, 2008 ▸; Janczak & Perpétuo, 2009 ▸; Marchewka *et al.*, 2003 ▸; Kapoor *et al.*, 2008 ▸; Bendjeddou *et al.*, 2009 ▸; Liang, 2010 ▸).

Les cations organiques (C_7_H_10_N)^+^ occupent des positions générales et assurent l’équilibre des charges négatives excédentaires portées par les anions [ClO_4_]^−^. La valeur moyenne de la liaison C—C dans le cycle aromatique est égale à 1,371 (8) Å. La distance C—N [1,451 (6) Å] dans le cation organique *p*-toluidinium est plus longue que la même distance dans le *p*-toluidine neutre [1,386 (4) Å; Ploug-Sørenson & Andersen, 1985 ▸], ceci est due à la protonation du groupement amine de la base organique *p*-toluidine. Les angles C—C—C et C—C—N sont compris entre 118,1 (5) et 121,6 (6)°, ils ne présentent pas d’anomalies et ils sont comparables à ceux du même cation associé avec d’autres types d’anions (Hosein *et al.*, 2011 ▸; Kapoor *et al.*, 2007 ▸; Rademeyer, 2005 ▸; Xu, 2010 ▸; Denne & Mackay, 1971 ▸; Denne *et al.*, 1971 ▸; Smirani *et al.*, 2004 ▸).

## Caractéristiques supra­moléculaires   

La cohésion structurale dans l’édifice cristallin est assurée par cinq liaisons hydrogène de type N—H⋯O établies entre les cations et les anions. Les distances et les angles décrivant les liaisons hydrogène sont donnés dans le Tableau 1 ▸.

**Figure 2:**
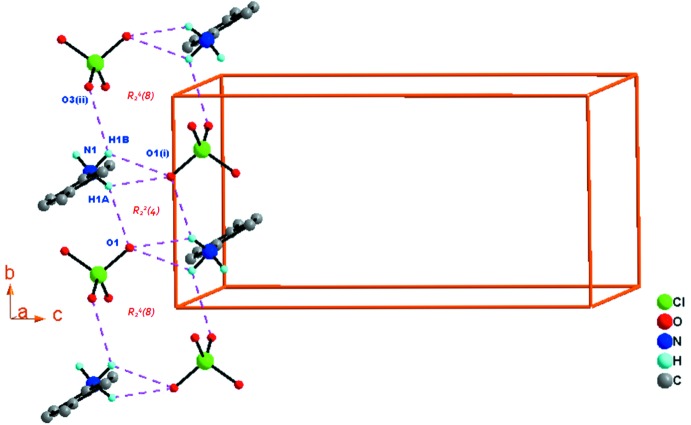
Une partie de la structure cristalline du composé (I) montrant la formation de la chaîne supra­moléculaire unidimensionelle, construite par les motifs 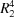
(8) et 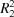
(4) s’étendant le long de direction [010].

**Figure 3:**
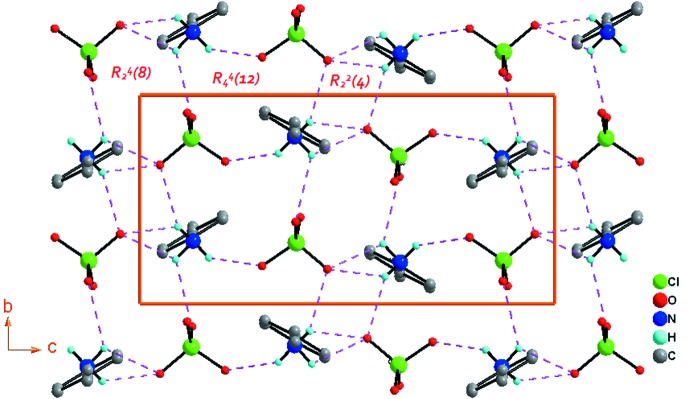
Projection selon la direction [100] d’une partie de la structure cristalline du composé (I) montrant la formation de la couche moléculaire, construite par les motifs 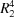
(8), 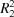
(4) et 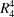
(12), parallèle au plan (100).

**Figure 4:**
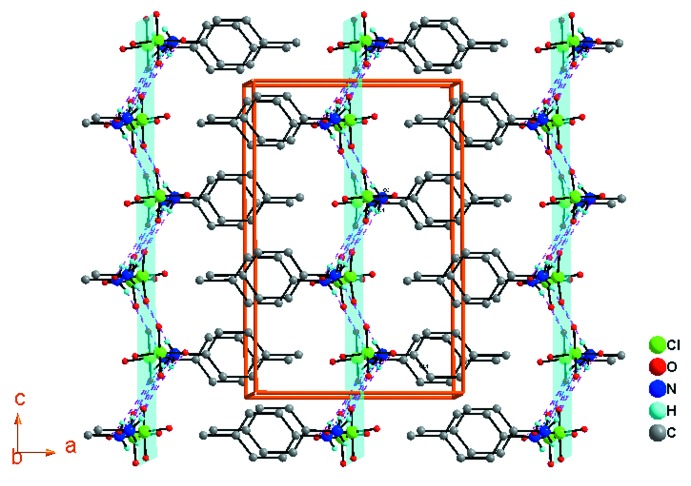
Vue en perspective de la structure cristalline du composé (I) montrant les couches moléculaires parallèles au plan (100).

Dans la structure du composé (C_7_H_10_N)[ClO_4_], les anions et les cations sont reliés par deux liaisons hydrogène N1—H1*B*⋯O1^i^ [code de symétrie: (i) −*x* + 1, −*y*, −*z* + 1] et N1-H1*B*⋯O3^ii^ [code de symétrie: (ii) − *x*, *y* + 1, *z*] pour former des motifs supra­moléculaires 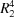
(8) autour du centre d’inversion cristallographique situé à (0, 0, *z*). Ces motifs 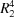
(8) positionnés sur l’unité de translation le long de la direction [010] sont aussi reliés par deux liaisons hydrogène N1—H1*A*⋯O1 et N1—H1*A*⋯O1^i^ pour former un nouveau motif supra­moléculaire supplémentaire de type *R*_2_^*2*^(4). Ces nouveaux motifs 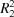
(4) occupent aussi le centre d’inversion cristallographique situé à (0, 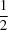, *z*). La translation infinie des deux motifs 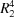
(8) et 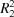
(4) construit une supermolécule sous forme d’une chaîne unidimensionnelle (1D) parallèle à la direction [010] (Fig. 2 ▸)*.* Les chaînes adjacentes reliées par l’axe 2_1_ sont connectées à travers la liaison hydrogène N1—H1*C*⋯O2^iii^ [code de symétrie: (iii) −*x* + 1, *y* + 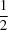, −*z* + 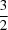] située sur l’axe 2_1_ le long (0, *y*, 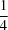), en donnant lieu au motif 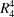
(12) (Fig. 3 ▸). Ces connections forment des couches bidimensionnelles d’anions et de cations construites à partir de motifs répétitifs 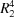
(8), 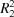
(4) et 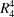
(12) s’étendant parallèlement au plan (100) (Fig. 3 ▸)*.* Une succession de couches moléculaires parallèles au plan (100) et situées à *x* = 2*n* + 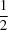 (*n* ∈ *Z*) est représentée dans la Fig. 4 ▸.

## Caractérisations par DRX sur poudre, IR et UV–Vis   

**Figure 5:**
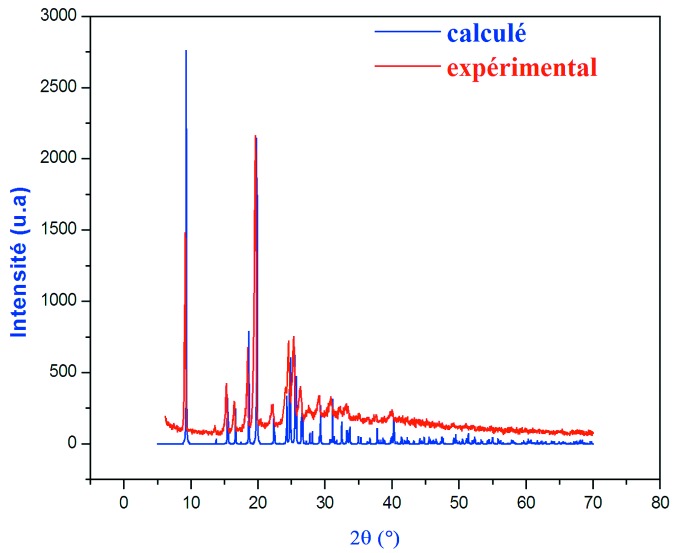
Diffractogrammes de poudre expérimental et calculé du composé (I).

La diffraction des rayons X sur poudre, à la longueur d’onde *K*_α_ du cuivre (λ= 1,5405 Å) du composé (I) a été effectuée au moyen d’un diffractomètre à deux cercles Bruker D8 Advance dans la gamme angulaire 8–70° en 2θ avec un pas de comptage de 0,02° et un temps de comptage de 1 seconde. Les diffractogrammes de poudre exérimental et calculé à l’aide du programme *PowderCell* (Kraus & Nolze, 1999 ▸) sont présentés dans la Fig. 5 ▸. La comparaison des deux difractogrammes montre sans ambiguïté la préparation du composé (C_7_H_10_N)[ClO_4_] à l’état pur.

**Figure 6:**
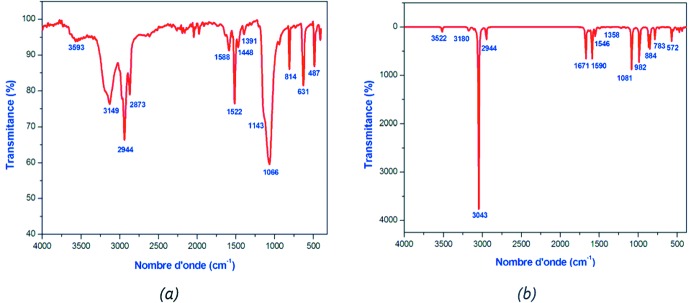
Spectres infrarouge expérimental (*a*) et théorique (*b*) du composé (I).

En se basant sur des études IR faites sur des composés ayant des similitudes avec le composé (C_7_H_10_N)[ClO_4_] (Drozd & Dudzic, 2013 ▸; Marchewka & Drozd, 2013 ▸; Ilczyszyn *et al.*, 2002 ▸; Anitha *et al.*, 2014 ▸; Erdogdu *et al.*, 2012 ▸), ainsi que les travaux de Nakamoto (Nakamoto, 1978 ▸), nous avons pu attribuer les bandes observées dans le spectre IR représenté dans la Fig. 6 ▸ *a* qui a été enregistré entre 400 et 4000 cm^−1^ en utilisant un spectromètre Perkin–Elmer où l’échanti­llon est dilué dans la poudre de KBr. Le Tableau 2 ▸ regroupe les résultats d’attribution et précise les activités en spectroscopie infrarouge du composé (I).

**Figure 7:**
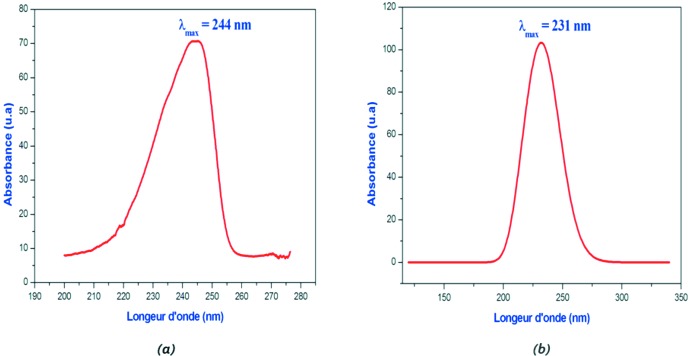
Spectres UV–Vis expérimental (*a*) et théorique (*b*) du composé (I).

Les mesures UV–Visible ont été effectuées au moyen d’un spectrophotomètre UNICO 2802 UV/VIS en utilisant une solution diluée de concentration *C* = 0,019 mol L^−1^. Le spectre UV–Vis obtenu du composé (I) est enregistré dans le domaine 200-800 nm en utilisant l’eau comme solvant (Fig. 7 ▸ *a*). Il exhibe une seule bande d’absorption vers λ_max_ = 244 nm attribuée à la transition π→π* dans le cycle aromatique du cation *p*-toluidinium.

## Calcul DFT   

**Figure 8:**
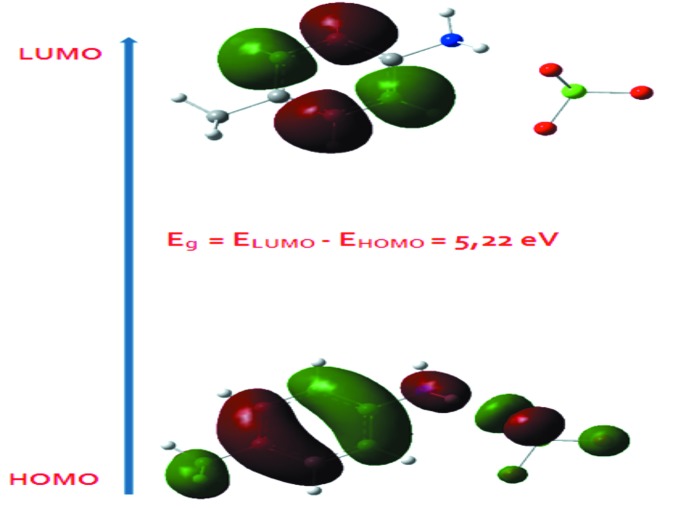
Distribution électronique des orbitales moléculaires LUMO et HOMO du composé (I).

Dans cette étude théorique, la fonctionnelle hybride B3LYP avec la base 6-311++G(d,p) (Becke, 1993 ▸) ont été utilisés dans tous les calculs faites par le programme Gaussian 09 (Frisch *et al.*, 2009 ▸). L’optimisation de la structure du composé étudié a été réalisée, à partir de la géométrie des rayons X. Une comparaison des résultats expérimentaux avec ceux théor­iques (Tableau 2) révèle que la plupart des valeurs calculées de longueurs et d’angles de liaisons sont très proches de celles expérimentales ce qui montre que le choix de la base 6-311++G(d,p) est convenable pour cette étude théorique, néanmoins la légère différence observée peut être attribuée au environnement de la molécule étudiée, étant isolé en phase gazeuse pour l’étude théorique et soumise à des inter­actions inter­moléculaires à l’état solide dans l’étude expérimentale. Le Spectre IR théorique du composé (I) est représenté dans la Fig. 6 ▸ *b* dont les valeurs de fréquences de vibration calculées ont été multiplié par le facteur de correction 0,97 (Tableau 3 ▸). Les résultats obtenus avec la méthode DFT [B3LYP/6-311++G(d,p)] sont en bon accord avec les resultats expérimentaux (Tableau 3 ▸). Le spectre UV théorique du composé étudié supposé à l’état gazeux a été calculé en utilisant l’approche TD–DFT (Fig. 7 ▸ *b*)*.* Il montre une seule bande d’absorption à λ_max_ égale 231 nm correspondant la transition π–π*. Le calcul théorique nous a permis aussi de calculer les énergies de l’orbitale moléculaire occupée la plus élevée HOMO (Highest Occupied Mol­ecular Orbital) et de l’orbitale moléculaire non occupée la plus basse LUMO (Lowest Unoccupied Mol­ecular Orbital). La distribution électronique des orbitales moléculaires HOMO et LUMO du composé étudié est représentée dans la Fig. 8 ▸. L’énergie de gap qui est la différence d’énergie des deux orbitales moléculaires précidentes (*Eg* = *E*_LUMO_ − *E*_HOMO_) vaut 5,22 eV.

## Analyse de surface Hirshfeld   

**Figure 9:**
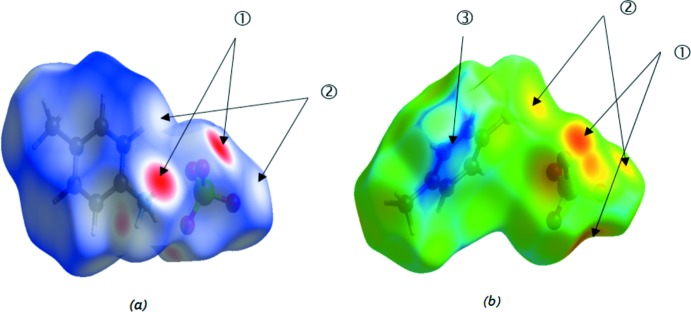
Surfaces Hirshfeld en modes *d*_norm_ (*a*) et *d*_e_ (*b*) du composé (I).

Pour avoir un aperçu sur la présence de liaisons hydrogène et des inter­actions inter­moléculaires dans la structure cristalline du composé (I), nous avons utilisé la surface Hirshfeld (Spackman & McKinnon, 2002 ▸; Spackman & Jayatilaka, 2009 ▸) et ses empreintes digitales bidimensionnelles qui sont calculés à l’aide du programme *CrystalExplorer* (Wolff *et al.*, 2012 ▸). Les surfaces Hirshfeld en modes *d*_norm_ et *d*_e_ sont illustrées dans les Fig. 9 ▸ *a* et 9*b*.

Dans la Fig. 9 ▸ *a* les tâches rouges identifiées par l’étiquette 1 correspondent aux contacts rapprochés de type H⋯O qui sont dus aux liaisons hydrogène N—H⋯O. Les zones blanches (étiquette 2) marquent les endroits où la distance séparant les atomes voisins avoisine la somme des rayons de van der Waals des atomes considérés, elles indiquent des inter­actions de type H⋯H. Les zones bleues illustrent les domaines où les atomes voisins sont trop éloignés pour inter­agir entre eux.

La surface Hirshfeld illustrée dans la Fig. 9 ▸ *b* est construite en employant *d*_e_ comme mode de représentation. Elle montre aussi l’existence des liaisons hydrogène de type N—H⋯O et des inter­actions H⋯H qui sont représentées par des tâches rouges entourées par des couronnes jaunes (étiquette 1) et des tâches jaunes (étiquette 2), respectivement. La couleur jaune indique que l’atome d’hydrogène de ces inter­actions est situé à l’intérieur de la surface Hirshfeld. L’emploi de la couleur bleue dans la surface identifiée par l’étiquette 3 indique que les contacts rapprochés dans cette région sont absents vu la grande distance inter­moléculaire séparant les atomes voisins.

**Figure 10:**
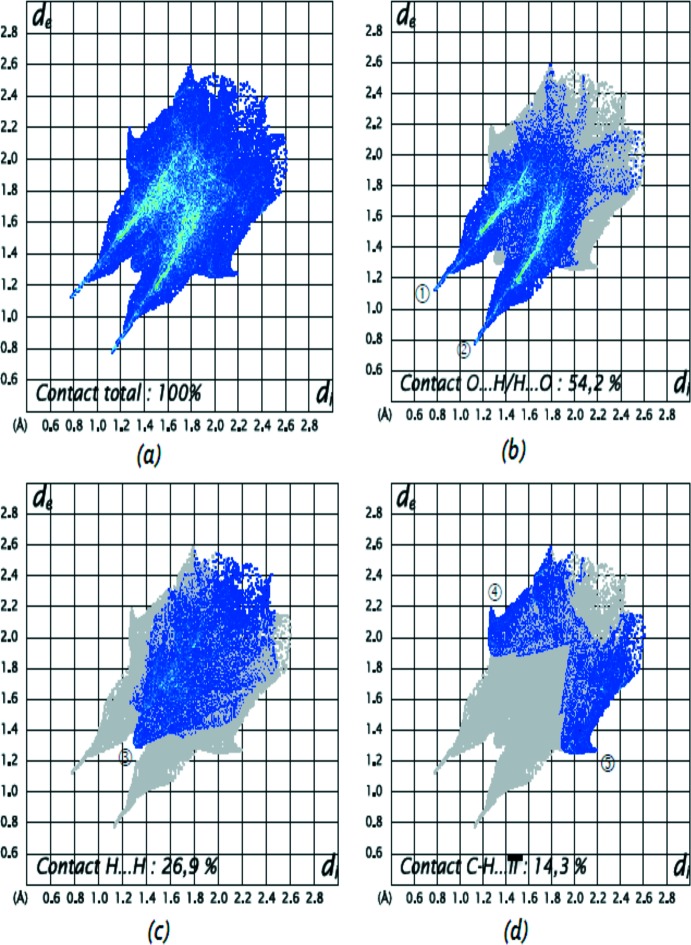
Empreintes digitales bidimensionelle du composé (I): tous les contacts inter­moléculaires (*a*), contacts O⋯H/H⋯O (*b*), H⋯H (*c*) et C—H..π (*d*).

La Fig.10*a* illustre l’empreinte bidimensionelle de la totalité des contacts contribuant à la surface Hirshfeld représentée en mode *d*_norm_. Le graphique exposé dans la Fig. 10 ▸ *b* représente les contacts H⋯O/O⋯H entre les atomes d’hydrogène situés à l’intérieur de la surface et les atomes d’oxygène situés à l’extérieur de la surface Hirshfeld et réciproquement. Il est caractérisé par deux pointes symétriques situées en haut et à gauche et en bas à droite avec *d*_e_ + *d*_i_ = 1,9 Å (étiquettes 1 et 2). Ces données sont caractéristiques des liaisons hydrogène N-H⋯O. Elles ont la contribution la plus importante à la surface Hirshfeld totale (54,2%).

Le graphique représenté dans la Fig. 10 ▸ *c* illustre l’empreinte bidimensionelle des points (*d*_i_, *d*_e_) associés aux atomes d’hydrogène (*r*_vdw_ = 1,20 Å). Il est caractérisé par une extrémité qui pointe vers l’origine et qui correspond à *d*_i_ = *d*_e_ = 1,3 Å (étiquette 3), ce qui révèle la présence des contacts rapprochés H⋯H au sein du composé étudié. Ces contacts H⋯H représentent 26,9% de la totalité de tous les contacts inter­moléculaires.

Le graphique présenté à la Fig. 10 ▸ *d* illustre les contacts entre les atomes de carbone situés à l’intérieur de la surface et les atomes d’hydrogène situés à l’extérieur de la surface Hirshfeld et réciproquement. L’analyse de ce graphique montre deux ailes symétriques du cotés gauche et droite (étiquettes 4 et 5). Ces données sont caractéristiques d’une inter­action de type C—H⋯π (14,3%).

La décomposition de l’empreinte digitale bidimensionelle montre aussi d’autres contacts: O⋯O (2,6%), C⋯C (1%) et C⋯O/O⋯C (1%).

## Synthèse et cristallisation   

Le composé (C_7_H_10_N)[ClO_4_] est obtenu en mélangeant dans l’eau, le *p*-toluidine C_7_H_9_N (pureté 99,6%, Sigma–Aldrich) et l’acide perchlorique (pureté 70%, Merck) selon les proportions molaire 1:1. Après agitation, la solution finale est laissée évaporer à température ambiante. Après cinq jours, des cristaux incolores sous forme de plaquettes commencent à apparaître. Ils ont une taille optimale pour une étude structurale.

## Affinement   

Les données cristallographiques, les conditions de la collecte et les résultats de l’affiniment de la structure du composé (I) sont regroupés dans le Tableau 4 ▸. Les atomes d’hydrogène liés aux atomes de carbone ont été fixés dans leurs positions géométriques calculés on appliquant les contraintes suivantes: C—H = 0,96 Å pour le groupement –CH_3_ avec *U*_iso_(H) = 1,5*U*_eq_(C) et C—H = 0,93 Å pour le groupement –CH avec *U*_iso_(H) = 1,2*U*_eq_(C). Les atomes d’hydrogène du groupement –NH_3_^+^ ont été determinés par synthèses de Fourier-différence et en fixant la distance *d*_N—H_ = 0,89 Å avec *U*_iso_(H) = 1,5*U*_éq_(N). Le cristal est maclé par mériédrie avec un angle β = 90.41°, émulant un système orthorhombique. Cette macle a été prise en compte en appliquant la matrice de macle [1 0 0, 0 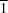 0, 0 0 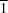] durant l’affinement de la structure.

## Supplementary Material

Crystal structure: contains datablock(s) I, publication_text. DOI: 10.1107/S2056989017018096/vm2207sup1.cif

Structure factors: contains datablock(s) I. DOI: 10.1107/S2056989017018096/vm2207Isup2.hkl

Supporting information file. DOI: 10.1107/S2056989017018096/vm2207Isup3.cml

CCDC reference: 1540997

Additional supporting information:  crystallographic information; 3D view; checkCIF report

## Figures and Tables

**Table 1:** Géométrie des liaisons hydrogène (Å, °)

*D*—H⋯*A*	*D*—H	H⋯*A*	*D*⋯*A*	*D*—H⋯*A*
N1—H1*A*⋯O1	0.89 (1)	2.26 (4)	3.011 (6)	142 (6)
N1—H1*A*⋯O1^i^	0.89 (1)	2.18 (5)	2.891 (6)	137 (6)
N1—H1*B*⋯O1^i^	0.89 (1)	2.44 (6)	2.891 (6)	112 (6)
N1—H1*B*⋯O3^ii^	0.89 (1)	2.42 (6)	2.959 (7)	120 (5)
N1—H1*C*⋯O2^iii^	0.89 (1)	2.08 (3)	2.882 (6)	150 (6)

Codes de symétrie: (i) 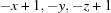; (ii) 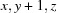; (iii) 
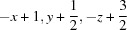
.

**Table 2:** Comparaison de quelques paramètres géométriques (Å, °) observés (X-ray) et calculés (DFT) du composé (I)

Paramètre	X-ray	B3LYP/6–311++G(d,p)			Paramètre	X-ray	B3LYP/6–311++G(d,p)
Cl1—O1	1,416 (4)	1,529			C1—C4	1,367 (7)	1,394
Cl1—O2	1,421 (4)	1,529			C2—C3	1,363 (7)	1,396
Cl1—O3	1,396 (4)	1,483			C2—C6	1,380 (8)	1,401
Cl1—O4	1,394 (5)	1,464			C2—C7	1,515 (7)	1,508
N1—C1	1,451 (6)	1,469			C3—C4	1,375 (7)	1,394
C1—C5	1,363 (7)	1,391			C5—C6	1,370 (8)	1,390
O4—Cl1—O3	110,6 (3)	112,69			C3—C2—C6	118,1 (5)	118,21
O3—Cl1—O2	110,1 (3)	108,72			C2—C3—C4	121,4 (5)	121,06
O1—Cl1—O2	105,3 (3)	105,94			C1—C4—C3	119,5 (5)	119,07
C5—C1—C4	120,1 (5)	121,54			C1—C5—C6	119,8 (5)	118,44
C5—C1—N1	119,3 (5)	118,67			C5—C6—C2	121,0 (5)	121,66

**Table 3:** Comparaison des fréquences expérimentales et calculées (cm^−1^) avec les attributions proposées du composé (I)

Fréquence expérimentale	Fréquence calculée	Attribution
3593; 3149	3416; 3084	υ_as_(–NH_3_^+^); υ_s_(–NH_3_^+^)
2944; 2873	2942; 2855	υ_as_(–CH_3_); υ_s_(–CH_3_)
1588; 1522	1620; 1542	δ_as_(–NH_3_^+^); δ_s_(–NH_3_^+^)
1448	1499	δ (–CH_3_)
1391	1317	υ(C—N)
1143; 1066	1048; 952	υ_as_ (ClO_4_^−^); υ_s_ (ClO_4_^−^)
814	857	υ(C—H)
631; 487	728; 554	δ_as_ (ClO_4_^−^) + δ_s_ (ClO_4_^−^)

**Table 4:** Détails expérimentaux

Données cristallines	
Formule chimique	C_7_H_10_N^+^·ClO_4_^−^
*M*_r_	207.61
Système cristallin, groupe d’espace	Monoclinique, *P*2_1_/*c*
Température (K)	293
*a*, *b*, *c* (Å)	9,513 (1), 7,196 (5), 14,297 (4)
β (°)	90,41 (2)
*V* (Å^3^)	978,7 (7)
*Z*	4
Type de rayonnement	Mo *K*α
μ (mm^−1^)	0,37
Taille du cristal (mm)	0,6 × 0,5 × 0,1

Collecte de données	
Diffractomètre	Enraf–Nonius CAD-4
Correction d’absorption	ψ scan (North *et al.*, 1968 ▸)
*T*_min_, *T*_max_	0,882, 0,998
Nombre de réflexions mesurées, indépendantes et observées [*I* > 2σ(*I*)] reflections	3216, 2128, 1160
*R*_int_	0,029
(sin θ/λ)_max_ (Å^−1^)	0,638

Affinement	
*R*[*F* ^2^ > 2σ(*F* ^2^)], *wR*(*F* ^2^), *S*	0,067, 0,212, 1,02
Nombre de réflexions	2128
Nombre de paramètres	129
Nombre de restraints	3
Traitement des atomes d’hydrogène	Atomes d’hydrogène traitées par un mélange d’affinement indépendant et contraint
Δρ_max_, Δρ_min_ (e Å^−3^)	0,36, −0,21

Programmes informatiques: *CAD-4 EXPRESS* (Duisenberg, 1992 ▸; Macíček & Yordanov, 1992 ▸), *XCAD4* (Harms & Wocadlo, 1995 ▸), *SHELXS97* (Sheldrick, 2008 ▸), *SHELXL2014* (Sheldrick, 2015 ▸), *DIAMOND* (Brandenburg, 2006 ▸), *WinGX* (Farrugia, 2012 ▸) et *publCIF* (Westrip, 2010 ▸).

## supplementary crystallographic information

### Crystal data

**Table:**

C_7_H_10_N^+^·ClO_4_^−^	*F*(000) = 432
*M**_r_* = 207.61	*D*_x_ = 1.409 Mg m^−^^3^
Monoclinic, *P*2_1_/*c*	Mo *K*α radiation, λ = 0.71073 Å
*a* = 9.513 (1) Å	Cell parameters from 25 reflections
*b* = 7.196 (5) Å	θ = 10–15°
*c* = 14.297 (4) Å	µ = 0.37 mm^−^^1^
β = 90.41 (2)°	*T* = 293 K
*V* = 978.7 (7) Å^3^	Plate, colorless
*Z* = 4	0.6 × 0.5 × 0.1 mm

### Data collection

**Table:**

Enraf–Nonius CAD-4 diffractometer	1160 reflections with *I* > 2σ(*I*)
Radiation source: fine-focus sealed tube	*R*_int_ = 0.029
Graphite monochromator	θ_max_ = 27.0°, θ_min_ = 2.1°
ω/2θ scans	*h* = −12→2
Absorption correction: ψ scan (North *et al.*, 1968)	*k* = −1→9
*T*_min_ = 0.882, *T*_max_ = 0.998	*l* = −18→18
3216 measured reflections	2 standard reflections every 120 reflections
2128 independent reflections	intensity decay: 1%

### Refinement

**Table:**

Refinement on *F*^2^	3 restraints
Least-squares matrix: full	Hydrogen site location: mixed
*R*[*F*^2^ > 2σ(*F*^2^)] = 0.067	H atoms treated by a mixture of independent and constrained refinement
*wR*(*F*^2^) = 0.212	*w* = 1/[σ^2^(*F*_o_^2^) + (0.085*P*)^2^ + 1.0514*P*] where *P* = (*F*_o_^2^ + 2*F*_c_^2^)/3
*S* = 1.02	(Δ/σ)_max_ < 0.001
2128 reflections	Δρ_max_ = 0.36 e Å^−^^3^
129 parameters	Δρ_min_ = −0.21 e Å^−^^3^

### Special details

**Table:**

Geometry. All esds (except the esd in the dihedral angle between two l.s. planes) are estimated using the full covariance matrix. The cell esds are taken into account individually in the estimation of esds in distances, angles and torsion angles; correlations between esds in cell parameters are only used when they are defined by crystal symmetry. An approximate (isotropic) treatment of cell esds is used for estimating esds involving l.s. planes.
Refinement. Refined as a two-component twin

### Fractional atomic coordinates and isotropic or equivalent isotropic displacement parameters (Å^2^)

**Table:**

	*x*	*y*	*z*	*U*_iso_*/*U*_eq_	
Cl1	0.52897 (14)	−0.29315 (14)	0.62359 (8)	0.0642 (4)	
O1	0.5256 (5)	−0.1659 (6)	0.5480 (2)	0.1049 (15)	
O2	0.5176 (5)	−0.1823 (6)	0.7054 (3)	0.1103 (15)	
O3	0.6554 (5)	−0.3917 (7)	0.6250 (4)	0.1262 (18)	
O4	0.4153 (6)	−0.4142 (7)	0.6138 (4)	0.147 (2)	
N1	0.6247 (5)	0.1992 (6)	0.6276 (3)	0.0721 (11)	
H1A	0.571 (6)	0.132 (8)	0.590 (4)	0.108*	
H1B	0.602 (7)	0.288 (7)	0.587 (4)	0.108*	
H1C	0.582 (6)	0.274 (7)	0.668 (3)	0.108*	
C1	0.7767 (5)	0.1822 (6)	0.6265 (3)	0.0589 (11)	
C2	1.0659 (6)	0.1509 (8)	0.6266 (4)	0.0756 (15)	
C3	0.9967 (6)	0.2379 (8)	0.5552 (4)	0.0809 (15)	
H3	1.0480	0.2862	0.5057	0.097*	
C4	0.8528 (6)	0.2557 (8)	0.5547 (3)	0.0755 (15)	
H4	0.8075	0.3174	0.5059	0.091*	
C5	0.8435 (6)	0.0943 (8)	0.6986 (4)	0.0827 (16)	
H5	0.7918	0.0449	0.7477	0.099*	
C6	0.9870 (6)	0.0790 (9)	0.6988 (4)	0.0896 (18)	
H6	1.0321	0.0193	0.7483	0.107*	
C7	1.2244 (6)	0.1306 (12)	0.6272 (6)	0.120 (3)	
H7A	1.2640	0.2097	0.6746	0.180*	
H7B	1.2606	0.1656	0.5673	0.180*	
H7C	1.2489	0.0038	0.6402	0.180*	

### Atomic displacement parameters (Å^2^)

**Table:**

	*U*^11^	*U*^22^	*U*^33^	*U*^12^	*U*^13^	*U*^23^
Cl1	0.0915 (9)	0.0434 (5)	0.0576 (6)	0.0046 (6)	−0.0020 (5)	0.0043 (6)
O1	0.179 (4)	0.069 (2)	0.067 (2)	−0.001 (3)	−0.005 (2)	0.0182 (18)
O2	0.163 (4)	0.098 (3)	0.069 (2)	0.022 (3)	0.016 (2)	−0.016 (2)
O3	0.124 (4)	0.099 (3)	0.155 (4)	0.050 (3)	−0.014 (3)	−0.011 (3)
O4	0.144 (4)	0.105 (4)	0.192 (6)	−0.052 (4)	−0.026 (4)	0.009 (4)
N1	0.071 (3)	0.059 (3)	0.086 (3)	0.002 (2)	−0.002 (2)	−0.014 (2)
C1	0.064 (3)	0.044 (2)	0.068 (3)	0.002 (2)	−0.005 (2)	−0.007 (2)
C2	0.070 (3)	0.071 (3)	0.086 (4)	0.004 (3)	0.003 (3)	−0.020 (3)
C3	0.079 (4)	0.086 (4)	0.079 (3)	−0.006 (3)	0.014 (3)	0.001 (3)
C4	0.082 (4)	0.076 (3)	0.068 (3)	0.007 (3)	−0.002 (2)	0.010 (3)
C5	0.085 (4)	0.083 (4)	0.081 (3)	−0.006 (3)	0.008 (3)	0.015 (3)
C6	0.085 (4)	0.086 (4)	0.097 (4)	0.014 (3)	−0.021 (3)	0.011 (3)
C7	0.073 (4)	0.144 (6)	0.143 (6)	0.020 (4)	−0.008 (4)	−0.050 (6)

### Geometric parameters (Å, º)

**Table:**

Cl1—O4	1.394 (5)	C2—C6	1.380 (8)
Cl1—O3	1.396 (4)	C2—C7	1.515 (7)
Cl1—O1	1.416 (4)	C3—C4	1.375 (7)
Cl1—O2	1.421 (4)	C3—H3	0.9300
N1—C1	1.451 (6)	C4—H4	0.9300
N1—H1A	0.885 (10)	C5—C6	1.370 (8)
N1—H1B	0.887 (10)	C5—H5	0.9300
N1—H1C	0.885 (10)	C6—H6	0.9300
C1—C5	1.363 (7)	C7—H7A	0.9600
C1—C4	1.367 (7)	C7—H7B	0.9600
C2—C3	1.363 (7)	C7—H7C	0.9600
			
O4—Cl1—O3	110.6 (3)	C2—C3—C4	121.4 (5)
O4—Cl1—O1	108.3 (3)	C2—C3—H3	119.3
O3—Cl1—O1	110.8 (3)	C4—C3—H3	119.3
O4—Cl1—O2	111.7 (4)	C1—C4—C3	119.5 (5)
O3—Cl1—O2	110.1 (3)	C1—C4—H4	120.3
O1—Cl1—O2	105.3 (3)	C3—C4—H4	120.3
C1—N1—H1A	121 (4)	C1—C5—C6	119.8 (5)
C1—N1—H1B	107 (4)	C1—C5—H5	120.1
H1A—N1—H1B	82 (5)	C6—C5—H5	120.1
C1—N1—H1C	121 (4)	C5—C6—C2	121.0 (5)
H1A—N1—H1C	118 (6)	C5—C6—H6	119.5
H1B—N1—H1C	83 (5)	C2—C6—H6	119.5
C5—C1—C4	120.1 (5)	C2—C7—H7A	109.5
C5—C1—N1	119.3 (5)	C2—C7—H7B	109.5
C4—C1—N1	120.5 (4)	H7A—C7—H7B	109.5
C3—C2—C6	118.1 (5)	C2—C7—H7C	109.5
C3—C2—C7	121.6 (6)	H7A—C7—H7C	109.5
C6—C2—C7	120.4 (6)	H7B—C7—H7C	109.5

### Hydrogen-bond geometry (Å, º)

**Table:**

*D*—H···*A*	*D*—H	H···*A*	*D*···*A*	*D*—H···*A*
N1—H1*A*···O1	0.89 (1)	2.26 (4)	3.011 (6)	142 (6)
N1—H1*A*···O1^i^	0.89 (1)	2.18 (5)	2.891 (6)	137 (6)
N1—H1*B*···O1^i^	0.89 (1)	2.44 (6)	2.891 (6)	112 (6)
N1—H1*B*···O3^ii^	0.89 (1)	2.42 (6)	2.959 (7)	120 (5)
N1—H1*C*···O2^iii^	0.89 (1)	2.08 (3)	2.882 (6)	150 (6)

Symmetry codes: (i) −*x*+1, −*y*, −*z*+1; (ii) *x*, *y*+1, *z*; (iii) −*x*+1, *y*+1/2, −*z*+3/2.
